# Dissemination Strategies From an HIV Self-Testing Intervention Among Adolescents and Young Adults in Nigeria: Crowdsourcing Open Call Study

**DOI:** 10.2196/89198

**Published:** 2026-08-04

**Authors:** Ucheoma Nwaozuru, Oluwakorede Adedeji, Yusuf Babatunde, Chisom Obiezu-Umeh, Lateef Blessing, Titilola Gbaja-Biamila, Folahanmi Akinsolu, Goodness Mirikwe, Thembekile Shato, Cassandra Akinde, Samantha Wu, Jarrel Clark, Temitope Ojo, Olufunto Olusanya, Oliver Ezechi, Joseph Tucker, Juliet Iwelunmor

**Affiliations:** 1 Wake Forest University School of Medicine Winston-Salem, NC United States; 2 University of Oxford Oxford, England United Kingdom; 3 University of Ilorin Ilorin, Kwara State Nigeria; 4 Northwestern University Chicago, IL United States; 5 Nigerian Institute of Medical Research Funder, Lagos Nigeria; 6 Wake Forest University Winston-Salem, NC United States; 7 Washington University in St. Louis St. Louis, MO United States; 8 University of Pennsylvania Philadelphia, PA United States; 9 University of North Carolina at Chapel Hill Chapel Hill, NC United States

**Keywords:** HIV, dissemination, crowdsourcing, adolescents and young adults, open call, digital platform, campaign, mobile app

## Abstract

**Background:**

Despite many evidence-based HIV interventions, most research findings are not disseminated. Enhancing research dissemination is essential to achieving Nigeria’s HIV targets.

**Objective:**

This study describes dissemination strategies solicited from the public to share research findings and products from an HIV self-testing intervention among Nigerian adolescents and young adults (AYAs).

**Methods:**

A crowdsourcing open call was launched in 2023 for 3 weeks to generate creative ideas for strategies to disseminate research findings and products from an HIV self-testing intervention among young people (aged 14-30 years). Crowdsourcing open calls are structured approaches to solicit community insights. Two people prescreened the submissions for eligibility before judging based on 5 criteria: clear description, desirability to AYAs, innovation, feasibility, and relevance to dissemination. We then conducted an inductive thematic content analysis of eligible submissions to identify key dissemination strategies for HIV research targeting AYAs and used descriptive statistics to characterize participants’ demographic data.

**Results:**

We received 64 eligible submissions from 24 states in Nigeria. Most (n=34, 53%) participants were male, and (n=18, 28%) were aged ≤18 years (mean age 21, SD 3.08 years). Our analysis identified six major themes reflecting AYA-driven strategies for disseminating HIV research products: (1) the use of digital platforms such as social media, websites, podcasts, and blogs; (2) the use of artistic expressions such as murals, posters, comics, and infographics to communicate key research findings in visually appealing ways tailored to AYAs; (3) community engagement and experiential activities such as door-to-door campaigns and interactive workshops to communicate research products and findings; (4) performance arts such as the use of concerts, open-mic events, dance performances, and fashion events to communicate research findings; (5) storytelling and narratives in the form of stories, drama, and theater as tools for dissemination; and (6) interactive and gamified activities such as mobile apps and board games to engage AYAs with research findings and products. Most submissions (39/64, 61%) recommended local collaboration with schools or community leaders for dissemination.

**Conclusions:**

The crowdsourcing open call was feasible and effective in generating ideas for research dissemination among AYAs. Our findings have implications for enhancing research dissemination among AYA populations in several resource-limited settings. Future work should involve evaluating the reach, acceptability, cost, and impact of these dissemination strategies.

## Introduction

Despite extensive progress and research focused on HIV prevention and treatment, especially among adolescents and young adults (AYAs; ages 14-30 years) [1,2], AYAs in low- and middle-income countries continue to face disproportionate HIV-related disparities, including higher transmission risks and poorer outcomes than older adults [3]. This implementation and practice gap is partly the result of ineffective dissemination of evidence about interventions that are known to improve health outcomes to intended end users, including AYAs [4]. Although dissemination is not a panacea for addressing this problem, it is useful for enhancing the uptake of evidence-based interventions and building trust among end users [5,6].

Dissemination, defined as the intentional and active process of spreading research evidence to target populations through determined channels and planned strategies [7,8], is essential to maximize the public health impact of research and research products. Researchers most commonly publish their findings in peer-reviewed journals, which serve as a benchmark for research excellence [9]. Although this channel has been effective in communicating research findings and products to researchers and communities with access to peer-reviewed journals, it has had limited reach among AYAs. This limitation undermines the reach and impact of findings and research products for AYAs [10-12]. Scientific evidence on the benefits of health research is most impactful when shared beyond academia [13,14].

Evidence shows a persistent gap in communicating research findings to study participants and the public [15,16]. This gap is particularly evident for AYAs in low- and middle-income countries, where limited attention has been given to innovative, AYA-centered dissemination strategies [4]. Few HIV studies have focused on research dissemination for AYAs [4]. Many HIV study findings are published in academic publications, which are often inaccessible to the AYAs who participate in studies and stand to benefit most from the research [10-12]. Beyond HIV research, the landscape of health research dissemination is highly fragmented, with a limited understanding of best practices for communicating research findings to diverse audiences [17]. Thus, bridging the persistent gap between scientific discovery and real-world application in HIV research will require innovative dissemination strategies that are accessible, equitable, and relatable to AYAs.

Crowdsourcing offers a participatory approach to coproducing research dissemination products that resonate with the public [18]. Crowdsourcing open calls solicit ideas from the public or from specific groups to solve a problem and then share the ideas back with the community [19]. These ideas are typically generated by end users, who can share insights from their lived experiences. This approach provides opportunities for end users to become more actively involved in solution generation. This may be particularly important for AYAs to ensure that dissemination strategies align with AYAs’ priorities and preferences [20]. In the context of AYA research, crowdsourcing has been a successful strategy for cocreating strategies that resonate with AYAs and can be leveraged to develop bottom-up strategies for disseminating research findings to AYAs [21,22].

This study used a crowdsourcing open call to identify innovative dissemination strategies for communicating research findings and products from the implementation of an evidence-based HIV self-testing (HIVST) intervention among AYAs in Nigeria—the Innovative Tools to Expand HIV Self-Testing (I-TEST) intervention (locally known as 4 Youth by Youth [4YBY]) [23,24]. Maximizing the public gains of this intervention would involve leveraging innovative dissemination strategies to communicate research products to end users, including AYAs themselves. This paper describes a crowdsourcing open call focused on generating ideas from AYAs for disseminating 4YBY research findings and products, and highlights AYA-generated dissemination strategies for communicating HIV research findings to AYAs in Nigeria.

## Methods

This study was conducted and reported in accordance with the COREQ (Consolidated Criteria for Reporting Qualitative Research) guidelines [25,26]. Refer to Multimedia Appendix 1 for the completed COREQ checklist.

### Description of the 4YBY Intervention

Briefly, the I-TEST intervention is an evidence-based intervention that leveraged crowdsourcing open calls, designathons, and apprenticeship training to cocreate intervention components and implementation strategies with AYAs to promote the reach and uptake of HIVST and other prevention services among AYAs in Nigeria [27]. Findings from the intervention showed an increase in the uptake of HIVST among Nigerian AYAs [27]. Details of this intervention have been described in previous publications [23,24].

### Overview of the Crowdsourcing Open Call

**Figure 1:**
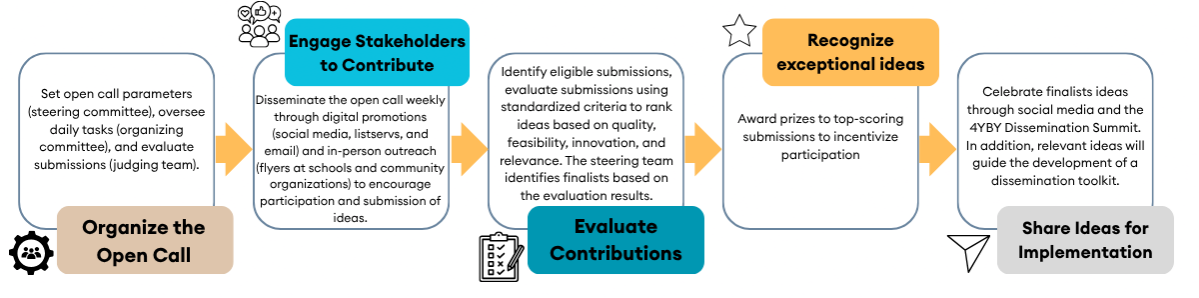
Steps in the crowdsourcing open-call process. 4YBY: 4 Youth by Youth.

Our research team launched a crowdsourcing open call from November 8, 2023, to November 29, 2023. The purpose of the open call was to solicit AYA-driven creative ideas for strategies to disseminate 4YBY research findings to AYAs in Nigeria. This included solutions that had been previously implemented, as well as those in the ideation phase. Eligible participants were between 14 and 30 years old and could submit ideas individually or as a team. The open call received entries for 3 weeks and was organized following the six-stage process outlined in the World Health Organization Special Programme for Research and Training in Tropical Diseases guide on crowdsourcing in health and health research: (1) identify problems, (2) establish governance for accountability, (3) engage the local community to submit ideas, (4) evaluate submissions, (5) recognize finalists, and (6) share and implement solutions [28] (Figure 1).

The open call was coordinated by the Nigerian Institute of Medical Research (NIMR) in partnership with the 4YBY team, which comprises researchers from Washington University in St Louis, Saint Louis University, Wake Forest University School of Medicine, and The University of North Carolina at Chapel Hill. A steering committee was convened to provide strategic advice and guidance throughout the open call process. The committee comprised diverse experts in HIV and community engagement from research institutions, government, media, industry, and AYA organizations. The open call was promoted through the 4YBY website, outreach via university student networks, youth-serving organizations, and partner institutions, as well as through social media platforms, including Instagram (Meta Platforms Inc), X (formerly Twitter; X Corp), Facebook (Meta Platforms Inc), and WhatsApp (Meta Platforms Inc).

### Data Collection

Submissions for the open call were collected using email, WhatsApp, and Google Forms. Participants were asked to submit entries addressing the prompt, “*How might we disseminate the research findings of 4YBY to AYA communities using creativity?*” The importance of using creative ways to share research findings with AYAs was emphasized on the open-call website. Submissions in response to the open-call prompt could be made in the form of text (maximum 500 words), images (maximum 5 MB), or videos (maximum 3 minutes). These were submitted via a Google Form on the 4YBY website, and participants submitting digital entries (images or videos) were instructed to email their files. Participants also had the option to submit their entries in a designated physical drop box at NIMR. In addition to responding to the open-call prompts, participants were asked to provide brief demographic information. For team submissions, demographic data were collected only for the team members who submitted the entry, not for all team members.

### Evaluation of Entries

Two members of the research team reviewed all submissions to determine eligibility. This initial screening ensured that each submission aligned with the open-call prompt. Once eligibility screening was concluded, submissions were judged based on five criteria: (1) clear and concise description; (2) desirability of the strategy to AYAs; (3) innovation of the proposed strategy; (4) feasibility of the solution for disseminating the research findings of 4YBY to other AYAs; and (5) relevance to dissemination (refer to Multimedia Appendix 2 for the judging rubric).

The judging team consisted of a panel of 5 experts in HIV, science communication, and local prevention services. Judges were provided with a judging rubric detailing the process, criteria, and procedures for declaring conflicts of interest. Each submission was assigned to all 5 judges, who independently scored the entries on a scale of 1 to 10 (1 being the lowest and 10 the highest) for each criterion, and a final mean score was calculated for each entry. After judging, the submissions with the highest scores were announced during the 9th Bioethics Conference and 4YBY Dissemination Summit, held from December 6, 2023, to December 8, 2023. The 3 highest-scoring entries were awarded monetary compensation of approximately US $632 (NGN 500,000), US $379 (NGN 300,000), and US $253 (NGN 200,000) for first, second, and third places, respectively. No additional incentives were offered beyond monetary prizes for the top 3 finalists.

### Data Analysis

Descriptive statistics were used to summarize and present the demographic characteristics of participants, including age, gender, and location. This provided a general overview of who took part in the open call. The qualitative data from the entries were analyzed using inductive thematic content analysis [29,30]. This involved systematically reviewing and coding the ideas submitted across different categories to identify recurring patterns, themes, and key messages. Identified concepts were categorized into themes and then applied to the deidentified crowdsourcing open-call textual data. Four members of the team (UN, GM, OA, and YB) independently coded submissions line by line, applying open coding to identify initial concepts. Codes were then discussed as a team and iteratively grouped into categories and themes through consensus. Thematic saturation was assessed iteratively during the check-in meetings, and thematic saturation was achieved when no new themes emerged. Identified concepts were categorized into themes and then applied to the deidentified crowdsourcing open-call textual data. The themes generated by this analysis provided insight into the creative expressions and perspectives shared by participants in response to the open-call prompt.

### AYA Engagement

AYAs were involved throughout all stages of the study, from the development of the crowdsourcing open-call prompt to the drafting of the final manuscript. AYAs served on the organizing committee for the crowdsourcing open call, contributing to the refinement of the open-call prompt and supporting dissemination efforts within their networks and on social media. In addition, 2 AYAs participated as judges in the evaluation of the open-call submissions, and 4 AYAs were involved in the analysis of the submissions and the codevelopment of this manuscript.

### Ethical Considerations

Ethics approval to conduct the research was received from the NIMR Institutional Review Board (project IRB 18-028). Electronic informed consent was obtained from all participants aged ≥18 years prior to participation in the crowdsourcing open call. The institutional review board granted a waiver of parental consent for participants aged 14 to 17 years due to the minimal-risk and nonsensitive nature of the crowdsourcing prompt, which involved generating creative ideas for research dissemination. Electronic assent was obtained from all participants aged <18 years in accordance with institutional review board guidance. Following the conclusion of the crowdsourcing open call, all submissions were deidentified to ensure participant anonymity. These procedures were implemented to uphold ethical standards and protect participant confidentiality throughout the research process.

## Results

### Summary of Participant Characteristics

We received 83 submissions to the open call over a 3-week period, of which 64 (77%) were eligible. Most entries (62/64, 97%) were submitted online via a Google Form on the study website, while approximately 3% (2/64) were submitted in person via a physical drop box at NIMR. Submissions were excluded if they were not responsive to the crowdsourcing open-call prompt or were duplicate entries. Of the 64 eligible submissions, 95% (n=61) were made by individual participants, while 5% (n=3) were submitted by groups. All solutions submitted to the crowdsourcing open call were in the idea phase, and none had been implemented by the time of submission.

Among the participants, 63% (40/64) were aged 19 to 24 years, 28% (18/64) were aged ≤18 years, and 9% (6/64) were aged >24 years. Gender distribution was nearly even, with 53% (35/64) identifying as men and 47% (31/64) identifying as women. Participants came from 24 states in Nigeria, with Rivers State accounting for the largest proportion of participants (9/64, 14%), followed by Oyo State (7/64, 11%) and Lagos State (6/64, 9%). Most participants (49/64, 77%) were unfamiliar with the 4YBY initiative prior to submitting their entries, while 23% (15/64) had previous knowledge of 4YBY programs. Additionally, 87% (56/64) were first-time applicants to the 4YBY crowdsourcing open-call contests, while 13% (8/64) had participated in previous 4YBY crowdsourcing open-call contests (Table 1).

**Table 1:** Key characteristics of participants in the 4 Youth by Youth dissemination crowdsourcing open call (N=64 eligible submissions).

Characteristics			Values
**Gender, n (%)**			
	Women	30 (47)	
	Men	34 (53)	
Age (years), mean (SD)			21 (3.08)
**Age group (years), n (%)**			
	15-18	18 (28)	
	19-24	40 (63)	
	25-30	6 (9)	
**Highest education completed, n (%)**			
	Junior secondary school	3 (5)	
	Senior secondary school	32 (50)	
	Some tertiary school	15 (23)	
	Bachelor’s degree	14 (22)	
**Geopolitical region, n (%)**			
	North Central	15 (23)	
	North East	2 (3)	
	North West	4 (6)	
	South East	5 (8)	
	South South	19 (30)	
	South West	19 (30)	

### Dissemination Strategies From the Crowdsourcing Open Call

**Figure 2:**
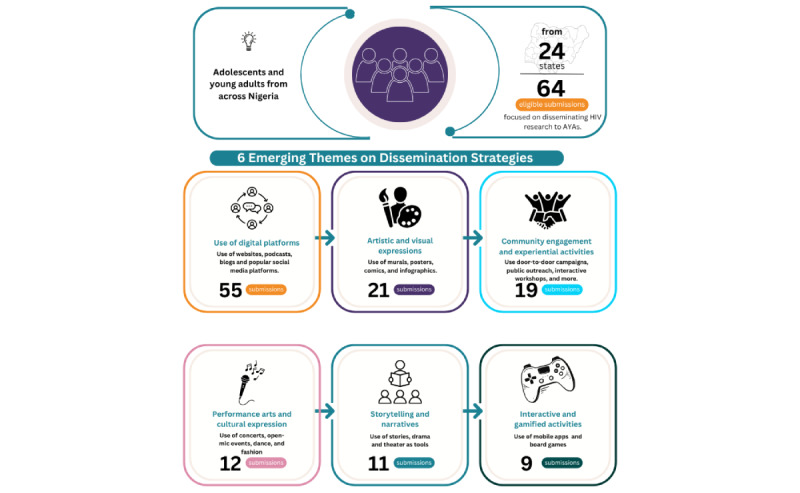
Summary of participants’ characteristics and emerging dissemination strategies from the 4 Youth by Youth dissemination crowdsourcing open call. AYA: adolescent and young adult.

Six major themes reflecting AYA-driven strategies for disseminating HIV research findings and products emerged from the crowdsourcing open call, as shown in Figure 2: (1) the use of digital platforms, (2) artistic and visual expressions, (3) community engagement and experiential activities, (4) performance arts and cultural expressions, (5) storytelling and narratives, and (6) interactive and gamified activities.

#### Theme 1: The Use of Digital Platforms

Most entries cited the use of digital tools (55/64, 86%) as a key dissemination strategy. The ideas included leveraging social media platforms (TikTok, Instagram, X, Facebook, and WhatsApp) frequently as a channel for communicating HIV research. Dissemination strategies included creating short, engaging videos to summarize research findings and launching social media hashtag campaigns to build momentum around the research. Some submissions suggested collaborating with social media influencers to communicate key research findings. These entries emphasized that social media platforms are accessible and engaging to AYAs and could potentially reach large audiences.

Beyond social media, some of the ideas included leveraging other digital tools, such as mobile apps and websites, as channels for dissemination. The ideas included developing mobile apps and websites that would serve as a clearinghouse for AYA HIV research and as platforms for communicating findings from AYA HIV research studies and HIV prevention studies. Blogs and podcasts were also recommended as accessible formats for sharing research findings in more engaging, relatable, and conversational ways. Other ideas included optimizing content for search engines to ensure visibility when AYAs search for HIV information online. In addition, radio was suggested as a dissemination channel for communicating key research findings through jingles and programming, reaching individuals who may not have access to the internet.

#### Theme 2: Artistic and Visual Expressions

The use of artistic and visual expressions emerged as the second most frequently mentioned dissemination strategy (21/64, 33%). Participants proposed using creative formats, such as murals, posters, comics, and infographics, to communicate key research findings in visually appealing ways tailored to AYAs. One participant suggested installing artistic expressions in public places, including schools and marketplaces, with the hope that they serve as conversation starters and help destigmatize HIV-related conversations. In addition, visually appealing and accessible infographics that simplify complex research findings were suggested as strategies for disseminating research findings.

#### Theme 3: Community Engagement and Experiential Activities

A total of 19 (N=64, 30%) responses focused on in-person community engagement and experiential activities as dissemination strategies for HIV research products. These included door-to-door campaigns and outreach in public spaces, including marketplaces, to communicate research products. Some of the ideas included having AYA-led seminars and campaigns delivered through these channels.

Interactive workshops and seminars were proposed as immersive, experiential learning experiences, with key findings communicated through discussions and collaborative content creation. One example included a school-based model in which workshops would deliver findings from AYA HIV research, combining in-person teaching with digital outreach.

#### Theme 4: Performance Arts and Cultural Expressions

Performance arts and cultural expressions were mentioned in 12 (19%) entries. Participants suggested using concerts, open-mic events, and dance performances as engaging platforms for disseminating research findings. Spoken words, rap, and poetry were also highlighted as creative formats for conveying key messages in ways that resonate with AYAs. One participant proposed hosting open-mic events in schools, lounges, community centers, and other spaces where AYAs naturally gather to spotlight research findings through what they called a “research slam” (spoken-word performances or poetry focused on research). Another idea included “sip and paint” sessions, where AYAs could express lessons learned from research through paintings.

Fashion was also identified as a potential medium for dissemination. Ideas included designing clothing embedded with messages conveying key research findings and serving as conversation starters to raise awareness about HIV prevention and AYA-focused research products.

#### Theme 5: Storytelling and Narratives

Eleven entries (17%) emphasized the role of storytelling and narratives, both fictional and real life, as dissemination strategies. Storytelling and narratives that included fictional stories, personal experiences, and character-driven plots were suggested as strategies for communicating research products to humanize HIV experiences, reduce stigma, and foster empathy. The stories shared in the submissions included personal stories of survival and resilience, as well as fictional narratives. One submission described a fictional story of an AYA who, after learning of a friend’s HIV diagnosis, mobilized a community outreach effort to promote testing and reduce stigma. Drama and theater were also highlighted as powerful storytelling tools, especially when tailored to local cultural contexts. Short films and documentaries based on research findings were also suggested as dissemination strategies for AYA HIV research products.

#### Theme 6: Interactive and Gamified Activities

A few submissions (9/64, 14%) suggested using interactive and gamified activities as a dissemination strategy. Specifically, educational games in the form of mobile apps, board games, or interactive simulations were suggested as strategies for conveying research findings to AYAs in fun and engaging ways. One submission suggested a mobile adventure game designed to educate players about HIVST through level-based missions informed by research evidence. Through quests and challenges, players would learn about HIV prevention, self-testing, and treatment options. In addition, gamification of these types of activities was suggested as a way to maintain attention, promote learning through interactive activities, and encourage peer-to-peer engagement.

## Discussion

### Principal Findings

We report innovative ideas proposed by AYAs to disseminate research products from an HIVST intervention. A crowdsourcing open call was able to solicit more than 80 entries in 3 weeks. This finding builds on existing literature highlighting the acceptability, feasibility, and potential of crowdsourcing open calls to engage communities in generating locally informed and relevant solutions [31-33]. The use of digital platforms, including a dedicated website and social media, was instrumental in reaching a diverse pool of participants, with approximately equal gender distribution and representation from all 6 geopolitical regions in Nigeria [34,35]. The findings from this crowdsourcing open call extend the literature by highlighting additional dissemination strategies that may resonate with and appeal to AYAs in Nigeria.

Our study’s findings suggest that adapting AYA research dissemination strategies is important. Research products need to be packaged in a way that is readily accessible and engaging to AYAs. This is consistent with other studies that emphasize the need for tailored, youth-relevant strategies for communicating research findings and products to AYAs [36]. The emergent themes included digital and in-person strategies, highlighting the importance of tailoring dissemination to AYAs’ preferences and lived experiences. These strategies reflect AYAs’ creativity and capabilities and offer alternatives to traditional academic dissemination formats. These dissemination strategies are not intended to replace conventional peer-reviewed publications but rather to provide complementary ways to communicate research products to broader audiences outside groups, such as researchers who may not find conventional academic publications most appealing [37]. This is consistent with other work that has highlighted the importance of making research widely accessible to end users by ensuring that it is easily comprehensible, engaging, and trustworthy [38].

The suggestion to use written creative pieces (including poetry, letters, storytelling, and creative nonfiction) was also common across multiple entries. Although researchers rarely use these creative pieces for research dissemination in Nigeria, these approaches offer a useful way to engage diverse communities and reduce cognitive burden. The current literature also supports the idea that poetry and storytelling are effective tools for sharing study findings with nonresearcher audiences in an impactful way [39,40]. Experiential learning via community events was another common theme. By demonstrating the use of HIVST in workshops, seminars, or other meetings, this dissemination strategy can help reach digitally excluded communities and build trust between communities and researchers. The use of art, whether in performance or display, for disseminating study evidence is not new globally but is less common in the local context. Traditionally, researchers have relied solely on journals and academic outlets for dissemination, without considering nonresearch audiences. However, the young population may require an alternative means of communication, and art offers value in this regard.

Consistent with emerging literature on dissemination strategies, social media platforms such as X (formerly Twitter), TikTok, and WhatsApp were identified as important channels for disseminating research evidence [41,42]. These platforms were envisioned as centralized hubs for AYA-focused HIV research, serving as platforms to share findings and promote awareness. Social media offers a potentially cost-effective way to communicate research products to the public [43] and has been successfully used during public health emergencies and outbreaks [44,45], including the COVID-19 pandemic [46,47]. In Nigeria, there is a growing body of work focused on understanding the role of social media in disseminating health-related information [48,49]. Given that social media is an appealing platform for AYAs, it may be beneficial to meet them where they are by leveraging these channels to communicate key research findings [50]. Some academic journals now have social media presences; however, few are designed to package information in ways that are digestible to the public, including AYAs. Other digital tools, such as podcasts, were also suggested. Podcasts have been shown to be effective for dissemination, thereby maximizing the utility of research outputs [51-53]. A recent scoping review on the use of podcasting as a research dissemination tool showed that podcasting provides an innovative, public-friendly space for researchers to share research evidence with the public [52]. As with other dissemination strategies, there is still a need to understand the reach and impact of podcasts in disseminating research products [52,54,55].

In addition, leveraging social media health influencers has also emerged as a potential strategy to communicate research findings to AYAs. Studies and reviews exploring the role of social media influencers—individuals on social media platforms who influence the behaviors and attitudes of their followers—for health communication have reported some promise [56,57]. This is particularly relevant in the Nigerian context, given the growing number of social media health influencers operating in the Nigerian social media space, including the likes of “Aproko Doctor,” “Flying Doctor,” and “Dr. Zobo,” with large followings [58,59]. Although social media health influencers may be an emerging avenue for disseminating research products, it is important to ensure that they provide accurate information, as concerns about inaccuracy or oversimplification have been identified as potential challenges associated with engaging them for public health communication [56,57]. Nonetheless, given the increasing influence of social media and social media influencers, it is important to develop strategies to leverage the broad reach of these platforms and influencers to disseminate research findings. Researchers can cocreate dissemination products with social media influencers and explore a colearning partnership in which researchers provide influencers with key research findings to ensure scientific accuracy, while influencers help researchers develop communication styles that are relatable to the public.

### Implications

The findings from this study have implications for research and practice. This crowdsourcing open call, like others among AYAs in Nigeria, demonstrates that AYAs are capable of generating creative and relevant solutions to issues that affect them [33,60]. These findings advance our understanding of public-informed strategies for dissemination and can inform how dissemination products are crafted to enhance their appeal among AYAs [61]. Innovative dissemination strategies, such as gamification, storytelling, poetry, and the use of digital tools, offer alternatives to traditional dissemination methods. However, further research is needed to assess the effectiveness, reach, acceptability, and cost-effectiveness of the proposed strategies, as all ideas identified in this study were in the concept phase and had not yet been tested. Nonetheless, this study provides ideas on dissemination strategies that may be appealing to AYAs.

Dissemination strategies typically consider who delivers the message, how it is shared, what it says, who it is intended for, and the setting in which it will be applied [62]. This study focused primarily on generating ideas about how the message is shared, but more research is needed on related elements. This area of inquiry might also explore the resources needed to execute these dissemination strategies, and the components that need to be adapted for context and audience. For resource-limited settings specifically, future work should examine which strategies can be implemented with minimal infrastructure; for example, radio, community outreach, and low-tech storytelling approaches may be more feasible starting points than mobile app development or large-scale concert events. Building partnerships with local universities, nongovernmental organizations, and government agencies may also help sustain dissemination efforts beyond the funding period. This may include specific training and resources to provide tailored dissemination strategies that meet the needs and preferences of various audience segments [15]. In addition, the cost-effectiveness of dissemination strategies is underexplored in the field [61]. Hence, there is a need for cost-related studies to better understand the costs of implementation and the cost-effectiveness of dissemination strategies.

### Limitations

Despite the strengths and implications of this work, these findings should be interpreted in light of some limitations. First, our small sample size may not fully reflect the opinions of Nigerian AYAs. Only data from 24 states were included in the open call, reducing the opportunity to capture diverse ideas from contextually different backgrounds. Only 3% (2/64) of submissions came from the North East region, which has among the highest HIV prevalence rates in Nigeria, while the South South and South West regions contributed a disproportionate 59% (38/64) of submissions. This skewed geographic distribution may have influenced the themes generated, particularly the strong emphasis on digital dissemination strategies, which may reflect the preferences of more urban and digitally connected participants rather than those of the broader AYA population. This may have been due to inadvertent exclusion based on the direction of the open-call announcements. Future crowdsourcing efforts should implement targeted outreach to underrepresented regions, including the North East.

In addition, participants’ demographic data suggested that we received relatively more submissions from AYAs with higher education. This was likely related to the framing of the open call and our stronger partnerships with universities and students. Nonetheless, in an attempt to reach a wider audience during the open call, we used infographics intended for a broader AYA audience. The use of online tools for open-call promotion also excludes people without internet access. To address this challenge, we offered participants the option to submit their ideas via a physical drop box at the NIMR in Lagos, which was mostly accessible to individuals residing in Lagos State or to those who had someone who could submit entries on their behalf. However, the low use of the drop box may reflect the geographic limitation of having a single submission site, suggesting the need to consider multiple physical submission points across and within states to enhance reach and accessibility.

In addition, participants’ prior experience with the 4YBY intervention was not required as an eligibility criterion, which may have affected the specificity of some submissions to the 4YBY context. Finally, as all submissions were in the ideation phase, no implementation or effectiveness data were available. Therefore, the strategies identified here are exploratory and should be understood as promising starting points for future empirical testing rather than validated best practices.

### Conclusions

This study demonstrates the creativity and insights of AYAs in cocreating dissemination strategies for HIV research products. This underscores the importance of engaging AYAs as active partners in issues that affect them and of ensuring that research products are packaged in ways that resonate with them. Engaging AYAs not only as audiences but also as cocreators of dissemination strategies can foster greater relevance and impact. Public-driven approaches can enhance dissemination efforts aimed at communicating research findings to a broader audience. By centering AYA voices, researchers and practitioners can move beyond traditional dissemination models and embrace innovative methods and integrate them into their dissemination plans. Future work should involve evaluating the feasibility, acceptability, cost, and impact of these dissemination strategies. Better research dissemination to AYAs can increase the translation of research findings into practice.

## Appendix

Multimedia Appendix 1 COREQ checklist.

Multimedia Appendix 2 Crowdsourcing Open Call Judging Rubric.

## Abbreviations

4YBY: 4 Youth By Youth
AYA: adolescent and young adult
COREQ: Consolidated Criteria for Reporting Qualitative Research
HIVST: HIV self-testing
I-TEST: Innovative Tools to Expand HIV Self-Testing
NIMR: Nigerian Institute of Medical Research

## Data Availability

The datasets generated and/or analyzed during this study are not publicly available due to privacy and confidentiality concerns but are available from the corresponding author on reasonable request.
