# Supplementary material for: Dissemination Strategies From an HIV Self-Testing Intervention Among Adolescents and Young Adults in Nigeria: Crowdsourcing Open Call Study
Source: JMIR Form Res. 2026 Aug 4;10:e89198. doi: 10.2196/89198 (PMC13487412; doi:10.2196/89198)
Supplement: Supplementary file 2 [file formative_v10i1e89198_app2.docx]

**Appendix File 2:** Crowdsourcing Open Call Judging Rubric

Thank you for serving as a judge for the 4Youth By Youth (4YBY) dissemination open call. We greatly appreciate your time and effort in supporting this initiative.

**Instructions:** You will be evaluating each submission based on the judging criteria below. Please provide a score on a scale of 1 to 10 (1 being the lowest quality and 10 the highest) for each criterion. During your evaluation, please focus on the content of the submissions. The total of each individual criterion will be computed. The scores for each submission will be tallied, averaged, and then ranked. We will provide a comments column for feedback*.*

Judging Criteria:

1. **Clear and concise description:** How clearly is the proposed dissemination strategy described? Were the details of the solutions clear and easy to understand?
2. **Desirability:** Will the proposed strategy be relevant for communicating research findings?
3. **Innovation of the proposed strategy:** How novel or innovative is the strategy?

How is the proposed solution new and different from what exists?

4. **Feasibility of the solution to disseminate 4YBY research findings/products to AYAs:** How practical or realistic is the solution? Is the strategy easily piloted? Are there metrics to evaluate the solution? Are there preliminary data or prototypes available?
5. **Relevance to dissemination:** Would the solution help in communicating research findings to AYAs? Is it aligned with the objectives of research communication?
